# Winsorization greatly reduces false positives by popular differential expression methods when analyzing human population samples

**DOI:** 10.1186/s13059-024-03230-w

**Published:** 2024-10-30

**Authors:** Lu Yang, Xianyang Zhang, Jun Chen

**Affiliations:** 1 Division of Computational Biology, Department of Quantitative Health Sciences, Mayo Clinic, Rochester, MN 55905 USA; 2 Center for Individualized Medicine, Mayo Clinic, Rochester, MN 55905 USA; 3 Department of Statistics, Texas A and M University, College Station, TX 77843 USA

## Abstract

A recent study found severely inflated type I error rates for DESeq2 and edgeR, two dominant tools used for differential expression analysis of RNA-seq data. Here, we show that by properly addressing the outliers in the RNA-Seq data using winsorization, the type I error rate of DESeq2 and edgeR can be substantially reduced, and the power is comparable to Wilcoxon rank-sum test for large datasets. Therefore, as an alternative to Wilcoxon rank-sum test, they may still be applied for differential expression analysis of large RNA-Seq datasets.

## Main text

A recent paper [1] reported that two popular differential expression analysis (DEA) tools, DESeq2 and edgeR, suffered from high false positive rates due to the violation of the negative binomial model assumption for real RNA-Seq data. The authors recommended the classical Wilcoxon rank-sum test for more robust differential expression analysis, at least, for large datasets. Their evaluation is rigorous and comprehensive, and the findings are thought-provoking. However, DESeq2 and edgeR have been the dominant DEA tools for almost a decade and it may not be easy to replace them with Wilcoxon rank-sum test in the near future. Compared to Wilcoxon rank-sum test, DESeq2 and edgeR are more interpretable and flexible. The coefficients obtained by these tools can be intuitively interpreted as log fold changes and covariate adjustment is natural in their framework. We thus wonder whether some preprocessing of the RNA-Seq data could improve the model fit of DESeq2/edgeR so that they can still be applied for DEA. As the authors showed that the main reason for the poor model fit for DESeq2/edgeR is the existence of outliers, and we also showed previously that the type I error inflation of DESeq2/edgeR could be substantially reduced by outlier replacement in microbiome data [2], we hypothesize that the type I error inflation of DESeq2/edgeR can also be controlled for RNA-Seq data using winsorization, an outlier replacement strategy that sets all outliers to a specified percentile of the data.

**Fig. 1:**
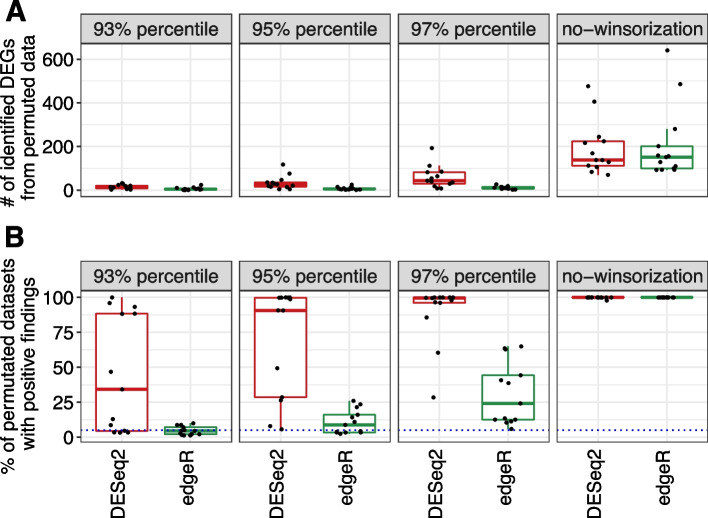
The effect of winsorization on false positive control of edgeR and DESeq2 based on permuted datasets. Different winsorization percentiles (93%, 95%, and 97%) were compared to no winsorization. Results were based on the averages from the 1,000 permuted datasets for the 13 population-level datasets. An FDR cutoff of 0.05 was used to identify DEGs. After winsorization, the average number of false DEGs on the permuted datasets (**A**) and the percentage of permuted datasets with any false findings (**B**) were significantly reduced. Each jittered point represents a dataset. The blue dotted line represents the 5% target FDR

We thus tested the hypothesis using the same 13 population-level RNA-Seq datasets from the original study [1]. To apply winsorization, we first normalized the count data by dividing the DESeq2 size factors (*estimateSizeFactors* function in R package “DESeq2”), and then for each gene, we replaced those normalized counts exceeding the $\alpha$ th percentile with the $\alpha$ th percentile ($\alpha =93, 95, 97)$. Finally, the winsorized normalized counts were multiplied by the size factors and rounded to the nearest whole number to produce winsorized counts. The winsorized count data were then used as the input to DESeq2/edgeR to obtain the p-values, followed by false discovery rate (FDR) control (Benjamini–Hochberg Procedure). We used the FDR cutoff of 5% to declare differentially expressed genes (DEG). We first studied the false positive control for DESeq2/edgeR after winsorization. As expected, winsorization greatly reduced the number of DEGs on the permuted datasets (Fig. 1A) and the percentage of permuted datasets with any positive findings (Fig. 1B). More aggressive winsorization resulted in better false positive control. With 93rd percentile winsorization, edgeR and DESeq2 identified on average 99.8% and 98.2% fewer DEG on the permuted datasets, respectively (Fig. 1A, edgeR range: 99.4–100%; DESeq2 range: 40.8–99.8%). The percentage of permuted datasets with any positive findings was also greatly reduced (Fig. 1B), and edgeR substantially outperformed DESeq2 in this measure. Under the null, the percentage of permuted datasets with any positive findings can be interpreted as the empirical FDR. edgeR was thus able to control the FDR near the target level of 5% when the data were winsorized at the 93rd percentile.

**Fig. 2:**
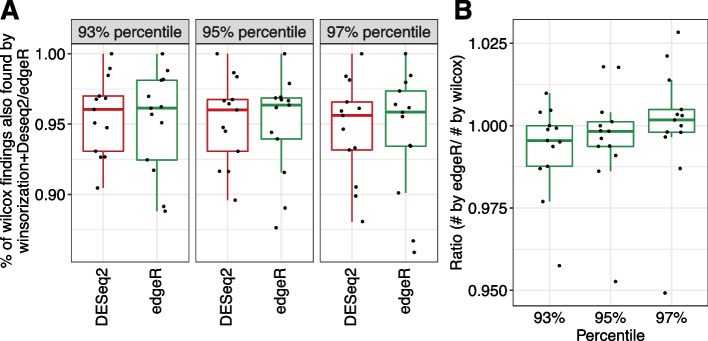
Comparison of the detected DEGs by edgeR and DESeq2 on the winsorized datasets to Wilcoxon rank-sum test. Different winsorization percentiles (93%, 95%, and 97%) were compared. An FDR cutoff of 0.05 was used to identify DEGs. **A** Percentage of DEGs detected by Wilcoxon rank-sum test also detected by edgeR and DESeq2 on winsorized datasets. **B** The ratio of the number of DEGs detected by edgeR on winsorized datasets to the number of DEGs by Wilcoxon rank-sum test. Each jittered point represents a dataset

We next studied the number of DEGs identified by DESeq2/edgeR on the non-permuted datasets after winsorization, comparing to the results based on the Wilcoxon rank-sum test, the recommended method by the original paper. We want to see whether the reduction in false positives is at the expense of power. We found that DESeq2 and edgeR were able to identify most of the DEGs detected by Wilcoxon rank-sum test after winsorization (Fig. 2A). DESeq2 had higher power than edgeR, however, the increased power could also be explained by its less efficient false positive control. We also compared the total numbers of DEGs identified by edgeR on the winsorized datasets to the numbers of DEGs by Wilcoxon. We found that edgeR had similar power as Wilcoxon rank-sum test using 93rd percentile winsorization, where edgeR could control the FDR close to the target level (Fig. 2B).

In summary, we demonstrated that the false positive rate for DESeq2/edgeR could be extensively reduced by winsorization. Compared to DESeq2, edgeR had better false positive control on the winsorized datasets. Thus, edgeR may still be used to perform DEA on large RNA-Seq datasets after winsorization. Based on the permutation results on real datasets, we recommend 95th percentile winsorization, which generally controls the FDR and retains most of the power. However, to rigorously select the percentile, the users may consider permuting the datasets many times and selecting the percentile that controls the number of false positives as we have done in Fig. 1.

## Data Availability

Data and codes for our study can be accessed via https://github.com/chloelulu/winsorization_GB2024 [3] under an MIT license. The version of the code used in this analysis is also available from Zenodo [4].
